# 18F-DOPA PET/CT and 68Ga-DOTANOC PET/CT scans as diagnostic tools in focal congenital hyperinsulinism: a blinded evaluation

**DOI:** 10.1007/s00259-017-3867-1

**Published:** 2017-11-08

**Authors:** Charlotte Dahl Christiansen, Henrik Petersen, Anne Lerberg Nielsen, Sönke Detlefsen, Klaus Brusgaard, Lars Rasmussen, Maria Melikyan, Klas Ekström, Evgenia Globa, Annett Helleskov Rasmussen, Claus Hovendal, Henrik Thybo Christesen

**Affiliations:** 1 Hans Christian Andersen Children’s Hospital, Odense University Hospital, Odense, Denmark; 2 Department of Clinical Research, University of Southern Denmark, Odense, Denmark; 3 Department of Nuclear Medicine, Odense University Hospital, Odense, Denmark; 4 Department of Pathology, Odense University Hospital, Odense, Denmark; 5 Department of Clinical Genetics, Odense University Hospital, Odense, Denmark; 6 Department of Abdominal Surgery, Odense University Hospital, Odense, Denmark; 7 Endocrine Research Centre, Moscow, Russia; 8 Astrid Lindgren Children’s Hospital, Karolinska Hospital, Stockholm, Sweden; 9 Ukrainian Center of Endocrine Surgery, Endocrine Organs and Tissue Transplantation, MOH of Ukraine, Kyiv, Ukraine; 10 Odense Pancreas Center (OPAC), Odense University Hospital, Odense, Denmark; 11 Department of Paediatrics, Odense University Hospital, Sdr. Blvd. 29, DK–5000 Odense C, Denmark

**Keywords:** Congenital hyperinsulinism, Hypoglycaemia, Positron emission tomography, Endocrine pancreas, Genetic diseases

## Abstract

**Purpose:**

Focal congenital hyperinsulinism (CHI) is curable by surgery, which is why identification of the focal lesion is crucial. We aimed to determine the use of 18F–fluoro-dihydroxyphenylalanine (18F-DOPA) PET/CT *vs*. 68Ga-1,4,7,10-tetraazacyclododecane-1,4,7,10-tetraacetic-acid-1-Nal3-octreotide (68Ga-DOTANOC) PET/CT as diagnostic tools in focal CHI.

**Methods:**

PET/CT scans of children with CHI admitted to Odense University Hospital between August 2005 and June 2016 were retrospectively evaluated visually and by their maximal standardized uptake values (SUV_max_) by two independent examiners, blinded for clinical, surgical and pathological data. Pancreatic histology was used as the gold standard. For patients without surgery, the genetic profile served as the gold standard.

**Results:**

Fifty-five CHI patients were examined by PET/CT (18F-DOPA *n* = 53, 68Ga-DOTANOC *n* = 18). Surgery was performed in 34 patients, no surgery in 21 patients. Fifty-one patients had a classifiable outcome, either by histology (*n* = 33, 22 focal lesions, 11 non-focal) or by genetics (*n* = 18, all non-focal). The predictive performance of 18F-DOPA PET/CT to identify focal CHI was identical by visual- and cut-off-based evaluation: sensitivity (95% CI) of 1 (0.85–1); specificity of 0.96 (0.82–0.99). The optimal 18F-DOPA PET SUV_max_ ratio cut-off was 1.44 and the optimal 68Ga-DOTANOC PET SUV_max_ cut-off was 6.77 g/ml. The area under the receiver operating curve was 0.98 (0.93–1) for 18F-DOPA PET *vs*. 0.71 (0.43–0.95) for 68Ga-DOTANOC PET (*p* < 0.03). In patients subjected to surgery, localization of the focal lesion was correct in 91%, and 100%, by 18F-DOPA PET/CT and 68Ga-DOTANOC PET/CT, respectively.

**Conclusion:**

18F-DOPA PET/CT was excellent in predicting focal CHI and superior compared to 68Ga-DOTANOC PET/CT. Further use of 68GA-DOTANOC PET/CT in predicting focal CHI is discouraged.

**Electronic supplementary material:**

The online version of this article (10.1007/s00259-017-3867-1) contains supplementary material, which is available to authorized users.

## Introduction

Congenital hyperinsulinism (CHI) is a rare, heterogeneous disease characterized by inappropriate insulin secretion from pancreatic islet β-cells resulting in hypoglycaemia [1]. The approximate incidence of persistent CHI is 1/40,000 live births in countries without founder mutations [2, 3]. The elevated serum insulin results in hypoketotic hypoglycaemia and a clinical presentation ranging from weak symptoms to loss of consciousness and seizures with a high risk of brain damage [1, 4].

The two major histological forms of CHI are the focal and the diffuse forms. Focal CHI occurs in 40–50% of cases [5] and is defined as a restricted pancreatic area with adenomatous β-cell hyperplasia, resulting in a lesion composed of confluent islets of Langerhans [6, 7]. Diffuse CHI involves the islets of Langerhans throughout the pancreas and is histologically characterized by hypertrophy of a few β-cell nuclei in most islets of Langerhans. In 50–66% of patients with diffuse CHI, mutations are found in the K_ATP_-channel genes *ABCC8* or *KCNJ11*, coding for the sulphonylurea receptor 1 (SUR1) and the potassium inward rectifier 6.2 (Kir6.2), respectively [6, 8]. Recessive inactivating mutations in *ABCC8*/*KCNJ11* are the most common causes of medically unresponsive diffuse CHI [9], whereas dominantly inherited *ABCC8/KCNJ11* mutations typically are medically responsive [1].

Focal CHI is strongly associated with a heterozygous, paternally inherited K_ATP_-channel germline mutation, as this combined with a somatic loss of heterozygosity in chromosome 11p15 in a focal region of the pancreas results in hemizygosity of the paternal *ABCC8*/*KCNJ11* mutation and loss of maternally expressed tumor suppressors [1].

The primary treatment goal is to increase and maintain blood glucose concentration above at least 3.5 mmol/L to avoid brain damage [10]. The treatment modalities include diet, i.v. glucose infusion, anti-insulin medical therapy (primarily diazoxide and octreotide) and pancreatic surgery [1]. Focal CHI is curable after a focal enucleation, or partial pancreatectomy, without subsequent risk of diabetes or malabsorption [9, 11]. Surgery for diffuse CHI should be restricted to medical non-responders and ranges from partial to subtotal pancreatectomy; the first is most often ineffective, and the latter imposes a high risk of diabetes mellitus and exocrine pancreatic insufficiency [11, 12].

Today, 18F–fluoro-dihydroxyphenylalanine (18F-DOPA) PET is the preferred tool in discerning focal from non-focal CHI [13]. L-DOPA is converted to dopamine by the aromatic amino acid decarboxylase enzyme in neuroendocrine cells [14]. The combined use of 18F-DOPA PET and CT [15–19] or MRI [20, 21] allows for a precise localization of the focal process prior to surgery. The preoperative identification of a focal lesion can be made by visual [15, 16, 18, 21] or quantitative [17, 19, 20, 22] methods. By the quantitative approach, a high standardized uptake value (SUV) ratio is used to identify the focal lesion. However, published diagnostic SUV ratio cut-offs range from 1.2 [22] to 1.5 [17, 19, 20]. 18F-DOPA is not available in every country and is more difficult to manufacture than another tracer, 68Ga-1,4,7,10-tetraazacyclododecane-1,4,7,10-tetraacetic acid-1-Nal3-octreotide (68Ga-DOTANOC). 68Ga-DOTANOC is a somatostatin analogue radiotracer with high affinity to the somatostatin receptor (SSTR) subtypes 2, 3, and 5. All the SSTR subtypes are variably expressed in endocrine cells of the islets of Langerhans [23], but the diagnostic value of 68Ga-DOTANOC PET/CT in the diagnosis of focal CHI is unknown [24]. As the waiting time to expert treatment is essential for the cerebral prognosis [4], a more easily available alternative to the 18F-DOPA PET would be desirable to shorten time to curative surgery for focal CHI patients born far from these centers.

We aimed to evaluate 18F-DOPA PET/CT and 68Ga-DOTANOC PET/CT as diagnostics tools in focal CHI preoperatively, analyzed visually or quantitatively, by ratio of maximal SUV values (SUV_max_ ratio) for 18F-DOPA PET/CT, or SUV_max_ for 68Ga-DOTANOC PET/CT.

## Patients and methods

We retrospectively investigated children with CHI admitted to the International Hyperinsulinism Center at Odense University Hospital, Denmark, between August 2005 and June 2016, subjected to 18F-DOPA PET/CT and/or 68Ga-DOTANOC PET/CT scan.

The CHI diagnosis was based on an inappropriately elevated p-insulin concentration during hypoglycaemia. Patients above 18 years were excluded, leading to final inclusion of 55 patients.

Anti-insulin medication was stopped two days prior to the PET/CT scan, allowing verification of the diagnosis of persistent CHI and evaluation of disease severity. To minimize stress for the patients and movement artifacts, 18F–DOPA PET/CT scans were obtained under general anesthesia (60-min acquisition time), while most 68Ga-DOTANOC PET/CT scans were performed under sedation (5-min acquisition time).

## PET/CT-based assessment

PET/CT scans were acquired on a GE Discovery PET/CT scanner (GE Medical System, Waukesha, WI, USA) and analyzed on a Dexus AW server 2.0. 18F–DOPA was produced by the electrophilic method. The patients were injected with 18F–DOPA or 68Ga-DOTANOC 4 MBq/kg, minimum 30 MBq. One field of view (FOV; acquisition time 5 min/FOV) was obtained over the pancreatic region at 10, 30 and 60 min. After injection, 68Ga-DOTANOC scans were obtained at 10, 30 and 60 min (*n* = 6), or at 30 min (*n* = 1), 45 min (*n* = 8) or 60 min (*n* = 3). A low-dose CT scan was performed prior to the first scan to be used for attenuation correction; acquisition parameters were 80–100 kV, 30–40 mA, rotation 0.8 s and pitch 0.984:1. A contrast-enhanced diagnostic CT scan over the pancreatic region was performed afterwards in all but one patient with suspicion of focal CHI to aid surgeons to localize the focal lesion: acquisition parameters were 120 kV, 30–400 mA, SmartmA, rotation 0,8 s, pitch 0.984:1 and noise index of 10. The CT scans were iteratively reconstructed to minimize radiation dose. Diagnostic CT scans were evaluated by radiologists.

PET/CT scans were anonymized and case numbers of 18F–DOPA, and 68Ga-DOTANOC scans were randomized independently from each other. SUV_max_ values were measured in g/ml and the region of interest (ROI) was shaped as a sphere. Results of the attenuation-corrected 18F–DOPA and 68Ga-DOTANOC PET/CT were evaluated by two independent researchers (C.D.C and A.L.N), blinded for all other clinical, radiological, surgical and pathological data. Disagreement was defined as a difference in SUV_max_ > 10%, a SUV ratio difference > 0.2 or a difference in visual or quantitative conclusion; the latter applied for 18F–DOPA PET based on a predefined SUV ratio cut-off = 1.45. Disagreements were resolved by a third party (H.P.). Two patients enrolled in the study were evaluated by A.T and M.H.V (see Acknowledgements) due to blinding issues, and disagreement was resolved by a third party (H.P).

The PET/CT scans were evaluated in the following order: 1) Visual method: a visually higher uptake of radiotracer in a part of the pancreas was considered positive for a focal process, and location was noted. A uniform uptake throughout the pancreas was considered diffuse. 2) Measurement of the SUV_max_ ratio in the pancreas: SUV_max_ in the part of the pancreas that visually appeared to be abnormal, divided by SUV_max_ in the part of the pancreas that visually appeared to be homogenous and normal (body or head). The SUV_max_ ratio was measured at 10, 30 and 60 min after injection. The highest SUV_max_ ratio was used and location was noted.

Patient files were evaluated after the blinded PET/CT evaluation. The following data were extracted from the medical records; sex, age at disease onset and at admission, country of origin, family history, genetic analyses, medication, maximal intravenous glucose infusion rate (off-medication prior to PET/CT scan) and lowest recorded blood glucose concentration. Insulin, proinsulin and C-peptide concentrations were obtained during hypoglycaemia, defined as a blood glucose below 2.5 mmol/L (neonatal period), or below 3.2 mmol/L (thereafter). The location and size of the lesions, results of frozen section microscopy and the final histological diagnoses were retrieved from the pathology report.

## Histopathological analysis

The histological diagnoses were retrieved from the files of the Dept. of Pathology, Odense University Hospital, Denmark, in all cases with surgery, except two patients who were operated upon abroad. For the final histological diagnoses, the pathology protocol for formalin-fixed, paraffin-embedded pancreatic tissue included hematoxylin-eosin of 4-μm-thick sections; immunohistochemical staining using the BenchMark Ultra immunostainer (Ventana Medical Systems, Tucson, AZ, USA) with the OptiView-DAB detection kit (Ventana Medical Systems, Tucson, AZ, USA); nuclear counterstaining with the BenchMark Ultra instrument using Hematoxylin II (Ventana Medical Systems, Tucson, AZ, USA), and coverslipping using a Tissue-Tek Film coverslipper (Sakura, Alphen aan den Rijn, The Netherlands). In most cases, immunohistochemical examination included synaptophysin, chromogranin A, insulin, glucagon, somatostatin, and the maternally expressed tumor suppressor p57. For preoperative frozen section analysis, 4-μm-thick frozen sections were stained with hematoxylin-eosin and tolouidin blue. Besides, particularly when the tissue specimens submitted for frozen section were small (< 5 mm), manual immunohistochemical staining of frozen sections for synaptophysin and insulin was performed.

## Genetic analysis

Prior to 2007, genetic analyses were performed by denaturing high-pressure liquid chromatography (dHPLC) analysis of *ABCC8* and *KCNJ11* as previously described [25]. A positive dHPLC was followed by Sanger sequencing [26]. From 2007 to 2013, analysis of *ABCC8*, *KCNJ11*, *GLUD1*, *GCK*, *HNF1-alfa* and *HNF4-alfa* was performed using Sanger sequencing [26]; from 2011, also including *HNF1-beta*, and from 2012, also including *HADH*, *MCT1* and *UCP2*. From 2013 and onwards, analysis of the before-mentioned genes was performed using next-generation sequencing (NGS) as previously described [27] followed by Sanger sequencing for confirmation of mutations found by NGS. Pathogenicity analyses were performed using multiple software programs [28–34]. Only previously reported mutations or rare DNA variants predicted by software analysis to be disease-causing were accepted as pathogenic.

## Statistics

Continuous variables were expressed by the median and interquartile ranges (IQR); categorical data in number and percentage. To estimate reproducibility, kappa values were calculated for categorical variables and intraclass correlation coefficients (ICC) for numerical variables. Reproducibility tests were made between patients analyzed by C.D.C and A.L.N.

Histopathology after surgery was used as the primary gold standard to discern focal from non-focal (diffuse or atypical) CHI. In patients not subjected to surgery, the absence of a heterozygous, paternal *ABCC8/KCNJ11* mutation was used as a secondary gold standard for non-focal CHI (no patients showed evidence of a dominant, paternal *ABCC8/KCNJ11* mutation). If no gold standard was available, the patient was excluded from analyses of test performance.

The performance of the PET/CT scans was tested on both the combined gold standard outcome (surgical and non-surgical patients) and on the primary gold standard outcome (histologically confirmed focal lesion after surgery).

To investigate test performance, receiver operating curves (ROC) were drawn from the SUV_max_ ratio (18F–DOPA PET) and the SUV_max_ (68Ga-DOTANOC PET). The optimal cut-off to maximize accuracy, the area under the curve (AUC) and the corresponding 95% CI were calculated by a bootstrap of 10,000.

Sensitivity, specificity, positive and negative predictive values (PPV and NPV, respectively) were calculated for the visual, or SUV-based, diagnostic prediction of focal CHI, including for the SUV ratio cut-off of 1.44 [21]. The 95% CIs were calculated by the Wilson method [35]. Comparison of SUV_max_ ratios and SUV_max_ values between groups was done using Mann–Whitney U tests. Comparison of PET/CT scan test performances was done by their 95% CI [36]. Comparison of superiority of ROC AUC of 18F–DOPA PET over 68Ga-DOTANOC PET was done using a non-paired bootstrap of 10,000.

Disease severity expressed as lowest blood glucose, or maximal glucose infusion demand without medication prior to PET/CT scan, was compared to the SUV_max_ ratio of 18F–DOPA PET in focal CHI by a linear model. In addition, the SUV_max_ ratio of the verified focal lesions were correlated to serum concentrations of insulin, proinsulin and C-peptide, and to the insulin-to-glucose ratio. Corresponding *p* values were calculated by *t* tests.

All data analyses were performed using the statistical software program R, version 3.1.2 [37], including several packages [38–44]. Level of significance was *p* < 0.05, trends 0.05–0.10.

## Results

Of the 55 children with CHI, 53 had a PET/CT scan with 18F–DOPA, and 18 had a 68Ga-DOTANOC scan, of which 16 also had an 18F–DOPA scan (Table 1). The median (IQR) age at PET/CT scan was 7 (3.5–18.5) months. Among patients who underwent surgery (*n* = 34), pathology reports showed focal CHI in 22 (64.7%), diffuse CHI in 10 (29.4%), atypical CHI in one (2.9%) and normal pancreatic histology in the analyzed tissue in one (2.9%; Table 2). The patients with non-focal CHI who underwent surgery were unresponsive to medication with high risk of (further) brain damage from hypoglycaemia. The operated upon patient with normal pancreatic tail histology and three patients without surgery had mutations of uncertain pathogenicity and were excluded from analyses of test performance and ROC.

**Table 1:** Patient characteristics

Patients	
Country of origin, no. (%)	
Belarus	2 (3.6)
Denmark and Greenland	13 (23.6)
Kazakhstan	3 (5.5)
Latvia	2 (3.6)
Norway	1 (1.8)
Russia	15 (27.3)
Sweden	10 (18.2)
Syria	1 (1.8)
Ukraine	7 (12.7)
United Kingdom	1 (1.8)
Sex	
Female (%)	25 (45.5)
Male (%)	30 (54.5)
Age at diagnosis (month), median (IQR)^a^, no. = 54	0 (0–0)
Age at PET/CT (month), median (IQR), no. = 55	7 (3.5–18.5)
Surgery, no. (%)	34 (61.8)
Disease severity, median (IQR)	
Lowest blood glucose (mmol/L), no. = 55	1.0 (0.6–1.6)
Glucose demand^b^ (mg/kg/min), no. = 51	8.5 (4.4–12.1)
P-insulin during hypoglycaemia (pmol/L)^d^, no. = 37	90 (48–115)
Insulin-to-glucose ratio^c^ (pmol/mmol) no. = 37	38.4 (18.1–55.6)
Genetic mutations, no. (%)	
*ABCC8*	34 (61.8)
Paternal	22
Maternal	1
Compound heterozygous	8
Homozygous	1
*De novo*	2
*KCNJ11*	4 (7.3)
Paternal	3
Maternal	1
*GLUD1*	1 (1.8)
*UPD11*	1 (1.8)
*HNF4-alfa*	1 (1.8)
No disease-causing mutations found	14 (25.5)

^a^Range: 0–9 months

^b^Medication-free

^c^Obtained during hypoglycaemia (glucose <2.5 mmol/L for age < 3 days, glucose <3.2 mmol/l for age ≥ 3 days)

^d^P-insulin at low p-glucose, reference <18 pmol/L

**Table 2:** Gold standard for type of CHI

	Number (%)
All patients	55
Included patients	51 (92.7)
Histology (after surgery): *n* = 33	
Focal	22 (64.7)
Non-focal	
Diffuse	10 (29.4)
Atypical	1 (2.9)
Genetics (no. surgery): *n* = 18	
Suspected focal^a^	0 (0)
Suspected non-focal	18 (85.7)
*ABCC8*: maternal, heterozygous	1
*ABCC8*: compound heterozygous	1
*ABCC8*: homozygous	1
*GLUD1*: heterozygous	1
*HNF4-alfa*: heterozygous	1
No mutations found	13
Excluded patients	4 (7.3)
Normal histology report	1 (2.9)
Genetic variants of uncertain pathogenicity	3 (14.3)
*ABCC8:* paternal	1
*ABCC8:* *de novo*	1
*KCNJ11:* maternal	1

^a^Heterozygous paternal *ABCC8*/*KCNJ11* mutation

**Fig. 1:**
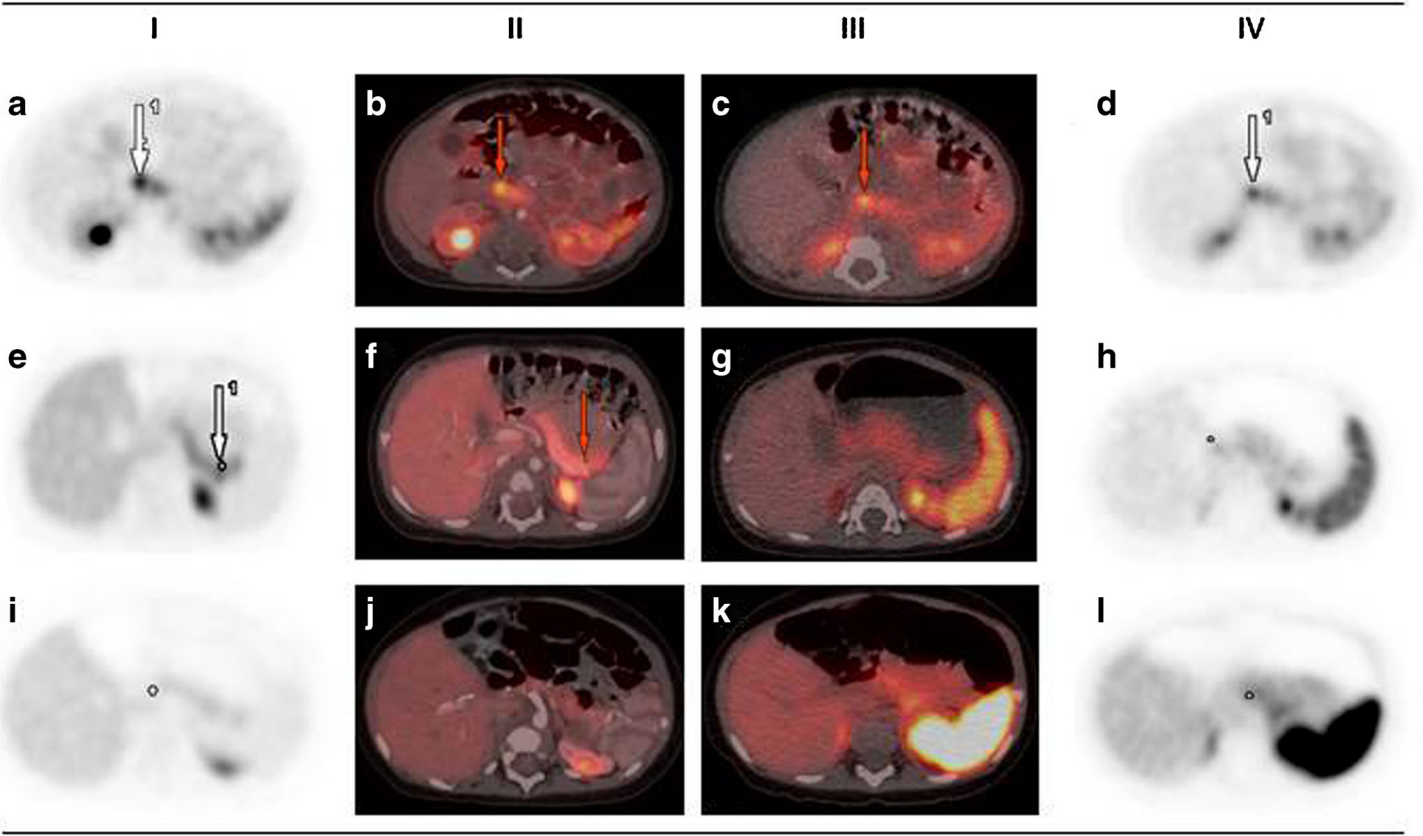
18F–DOPA PET/CT and 68Ga-DOTANOC PET/CT performed in three patients. **a**–**d** Patient 1, focal CHI. **e**–**h** Patient 2, focal CHI. **i**–**l** Patient 3, diffuse CHI. Column I shows 18F–DOPA PET; column II 18F–DOPA PET/CT (here contrast-enhanced); column III 68Ga-DOTANOC PET/CT; column IV 68Ga-DOTANOC PET. Red arrows point to the focal lesions. In the blinded evaluations, patient 1 and 2 were correctly diagnosed as focal and patient 3 as non-focal CHI by 18F–DOPA PET/CT (visual and by cut-off method). By 68Ga-DOTANOC PET/CT, patient 1 and patient 3 were correctly classified, but patient 2 was wrongly classified as non-focal

Examples of 18F–DOPA PET/CT and 68Ga-DOTANOC PET/CT are shown in Fig. 1.

The kappa value for the visual evaluation of the 51 18F–DOPA PET/CT scans was 0.74 (95% CI 0.55–0.93) and 0.89 (0.68–1) for the 18 68Ga-DOTANOC PET/CT. The ICC of the SUV_max_ ratio values of the 18F–DOPA PET scans was 0.96 (0.94–0.98). For 68Ga-DOTANOC PET, the ICC of the SUV_max_ values was 0.95 (0.88–0.98). Additional ICC values for 68Ga-DOTANOC PET are shown in Online Resource Table 1.

## Evaluation of 18F–DOPA PET/CT

Visual evaluation of 18F–DOPA PET/CT showed a sensitivity of 1 (0.85–1), specificity of 0.96 (0.82–0.99), a PPV of 0.96 (0.79–0.99) and an NPV of 1 (0.87–1) for the prediction of focal CHI (Table 3a). Equivalent results were obtained when only histology was used as the isolated gold standard (Table 3b). Tracer uptake in the gall bladder and common bile duct was seen once at the 10-min series and more frequently at 30 or 60 min. The variable staining allowed discrimination of common bile duct staining from suspected focal lesion.

**Table 3:** Test performance of 18F–DOPA PET/CT and 68GA-DOTANOC PET/CT in predicting focal CHI

Method	No.	Sensitivity (95% CI)	Specificity (95% CI)	PPV (95% CI)	NPV (95% CI)	Location^a^ (%)
a) Patients with histology or genetics as gold standard						
18F–DOPA PET visual	49	1 (0.85–1)	0.96 (0.82–0.99)	0.96 (0.79–0.99)	1 (0.87–1)	20/22 (91)
18F–DOPA PET cut-off 1.44	49	1 (0.85–1)	0.96 (0.82–0.99)	0.96 (0.79–0.99)	1 (0.87–1)	20/22 (91)
68Ga-DOTANOC PET visual	16	0.78 (0.45–0.94)	0.86 (0.49–0.97)	0.88 (0.53–0.98)	0.75 (0.41–0.93)	7/7 (100)
68Ga-DOTANOC PET cut-off 6.77	16	0.67 (0.35–0.88)	0.71 (0.36–0.92)	0.75 (0.41–0.93)	0.63 (0.31–0.86)	6/6 (100)
b) Patients with histology as gold standard						
18F–DOPA PET visual	32	1 (0.85–1)	1 (0.72–1)	1 (0.85–1)	1 (0.72–1)	20/22 (91)
18F–DOPA PET cut-off 1.44	32	1 (0.85–1)	1 (0.72–1)	1 (0.85–1)	1 (0.72–1)	20/22 (91)
68Ga-DOTANOC PET visual	14	0.78 (0.45–0.94)	0.80 (0.38–0.96)	0.88 (0.53–0.98)	0.67 (0.30–0.90)	7/7 (100)
68Ga-DOTANOC PET cut-off 7.73	14	0.56 (0.27–0.81)	0.60 (0.23–0.88)	0.71 (0.36–0.92)	0.43 (0.16–0.75)	5/5 (100)

^a^Correctly located/correct focal identification according to histology after surgery

PPV, positive predictive value, NPV, negative predictive value

The maximal tracer values by 18F–DOPA PET were obtained at 10 min in 12 patients (focal *n* = 4), at 30 min in 13 patients (focal *n* = 5) and at 60 min in 24 patients (focal *n* = 13). SUV_max_ ratio values of the focal lesions had a median (IQR) of 1.72 (1.58–2.27), ranging from 1.47 to 4.69, compared to 1.12 (1.05–1.20), with a range of 0.94–1.73, for the non-focal type *(p* < 0.0001). The classified non-focal patient with an SUV_max_ ratio of 1.73 was not subjected to surgery and had a normal *ABCC8*/*KCNJ11* genetic analysis, which may represent a false negative genotype for focal CHI.

**Fig. 2:**
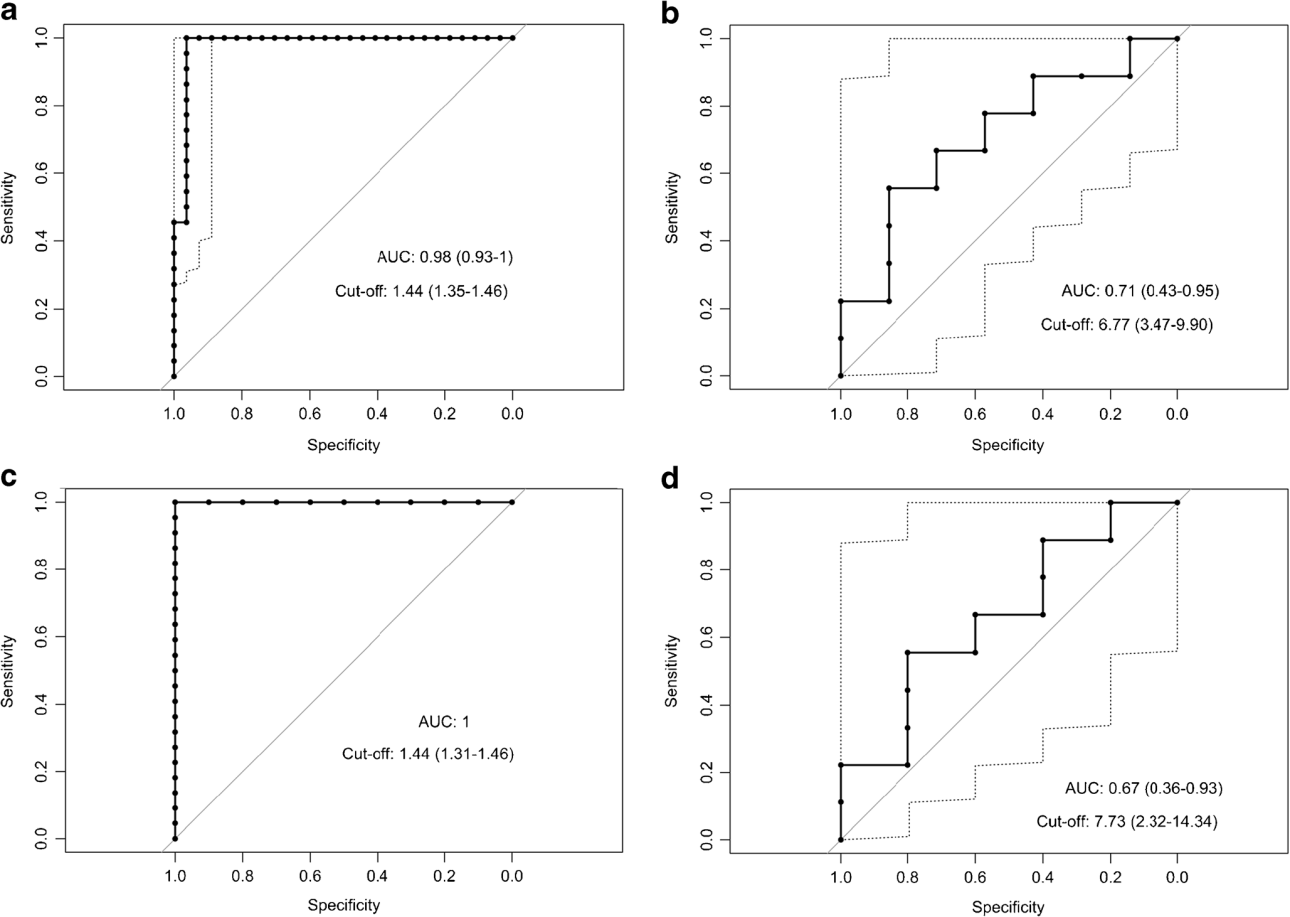
ROC curves for the performance of PET/CT scans in predicting focal CHI. **a** 18F–DOPA PET, SUV ratio, **b** 68Ga-DOTANOC PET, SUV_max_, **c** 18F–DOPA PET, SUV ratio with histology as the singular gold standard, **d** 68Ga-DOTANOC PET, SUV_max_ with histology as the singular gold standard

The performance of 18F–DOPA PET was excellent with an ROC AUC of 0.98 (0.93–1; Fig. 2a). The optimal SUV_max_ ratio cut-off was 1.44 (1.35–1.46). Equivalent results were obtained when using histology as the isolated gold standard (Fig. 2c).

Quantitative evaluation of 18F–DOPA PET using the cut-off of 1.44 showed the exact same estimates for test performance as the visual evaluation (Table 3).

## Evaluation of 68Ga-DOTANOC PET/CT

Visual evaluation of 68Ga-DOTANOC PET/CT showed a test performance with a sensitivity of 0.78 (0.45–0.94), a specificity of 0.86 (0.49–0.97), a PPV at 0.88 (0.53–0.98) and an NPV at 0.75 (0.41–0.93). Equivalent results were obtained when histology was used as the isolated gold standard (Table 3).

The maximal tracer values by 68Ga-DOTANOC PET were obtained in 30–60 min (zero patients at 10 min, two patients at 30 min; focal *n* = 1), seven patients at 45 min (focal *n* = 3) and seven patients at 60 min (focal *n* = 5). SUV_max_ values of the focal lesions had a median (IQR) of 9.43 (5.86–12.51) with a range of 2.32–16.81 g/ml, compared to 4.71 (3.27–6.98) with a range 2.31–13.42 g/ml for the non-focal type (*p* = 0.17).

The diagnostic performance of SUV_max_ in 68Ga-DOTANOC PET was fair with an ROC AUC of 0.71 (0.43–0.95; Fig. 2b). The optimal SUV_max_ cut-off was 6.77 (3.47–9.90) g/ml. Test performance showed a sensitivity of 0.67 (0.35–0.88), a specificity of 0.71 (0.36–0.92), a PPV of 0.75 (0.41–0.93) and an NPV of 0.63 (0.31–0.86). No significant difference was obtained if applying histology only as the gold standard (Fig. 2d, Table 3).

Differences between point estimates of the visual analysis vs. SUV_max_ cut-offs by 68Ga-DOTANOC PET did not reach significance.

## DOPA *vs*. DOTANOC scans

The ROC AUC of 18F–DOPA PET showed a superior performance compared to 68Ga-DOTANOC PET; *p* = 0.025 (histology and genetic analysis as the gold standard), and *p* = 0.017 (histology only as the gold standard).

The 18F–DOPA PET/CT (by visual or SUV ratio) detected the correct location in 91% (20/22) of the cases with focal lesions. Two lesions were incorrectly located. The first patient with an incorrectly located focal lesion (from 2007, no supplementary 68Ga-DOTANOC PET scan and no diagnostic CT scan) was deemed to be located in the head of the pancreas; the lesion was later identified at the tip of the pancreatic tail. The second patient (from 2016, no supplementary 68Ga-DOTANOC PET scan) had an ectopic lesion in the duodenum near the major duodenal papilla. The lesion was horseshoe-shaped and extended into the pancreas. The pancreatic part of the lesion was correctly located by the PET/CT but the true nature of the lesion was classified by histology.

The 68Ga-DOTANOC PET/CT (visual and SUV_max_) scans detected the correct location in 7/7 and 6/6 of the cases.

## 68Ga-DOTANOC PET SUV_max_ ratios

In a post-hoc analysis of 68Ga-DOTANOC PET, we attempted calculating SUV_max_ ratios by use of normal pancreatic tissue, spleen or liver as reference. The resulting ROC curve and test values were not significantly different from the use of pancreatic SUV_max_. Data are given in Online Resource Table 2 and Online Resource Fig. 1.

## Disease severity and SUV_max_ ratio

**Fig. 3:**
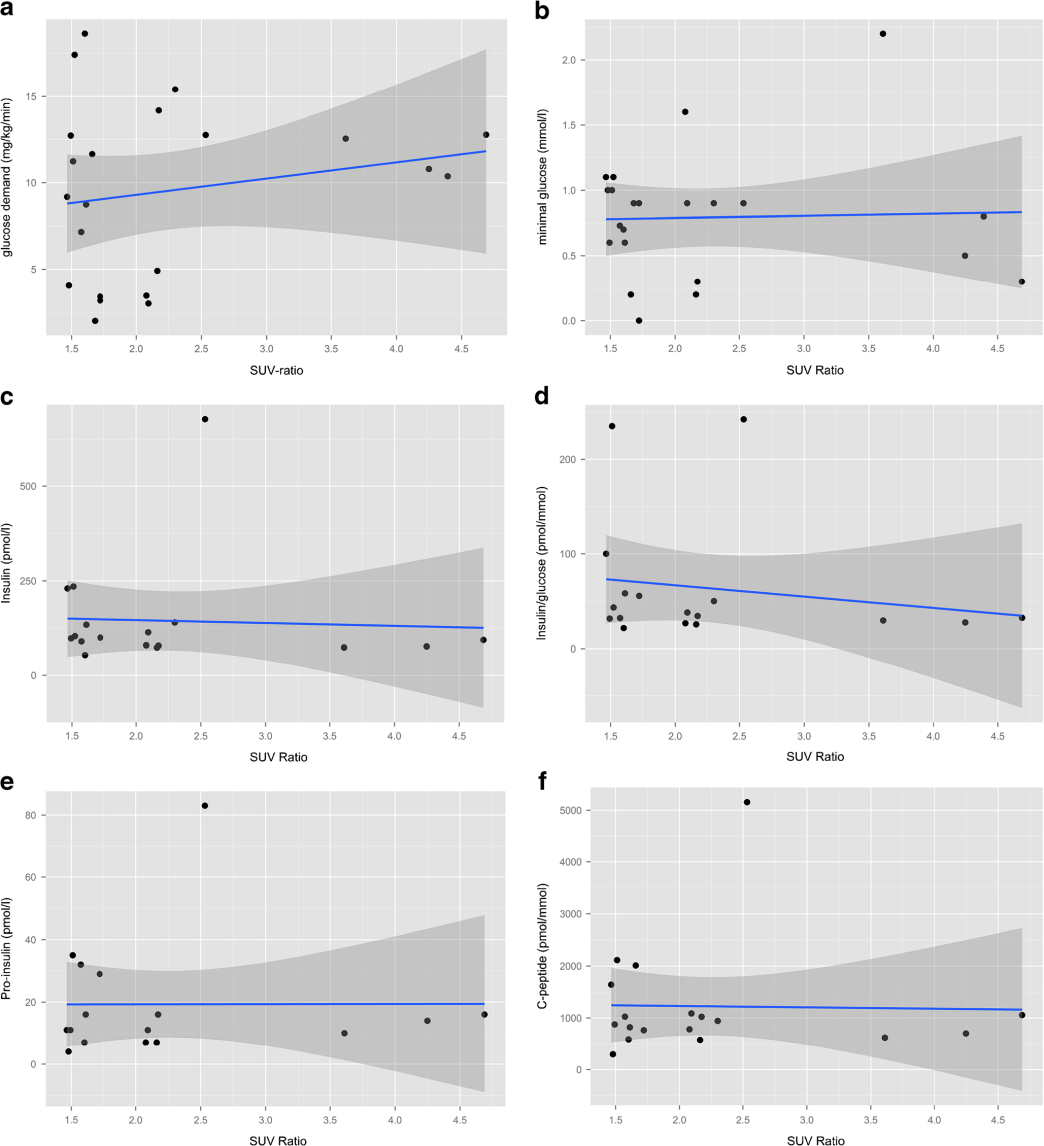
Correlation of focal SUV ratio and disease severity. **a** Intravenous glucose demand, **b** minimal glucose value, **c** insulin concentration (pmol/L), **d** insulin-to-glucose ratio (pmol/L: mmol/L), pro-insulin concentration (pmol/L) and **e** C-peptide concentration (pmol/L). The gray area indicates the 95% CI for the linear model

No relationship was observed between the minimal blood glucose, or maximal glucose infusion demand, and the 18F–DOPA PET SUV_max_ ratio of a focal lesion (*p* = 0.88 and *p* = 0.39, respectively; Fig. 3a–b). Equivalent results were obtained when analyzing serum insulin, C-peptide, proinsulin and insulin-to-glucose ratios (Fig. 3c–f).

## Discussion

In this blinded retrospective study, we found significantly better performance of 18F–DOPA PET/CT compared to 68Ga-DOTANOC PET/CT in preoperative prediction of focal CHI. For 18F–DOPA PET/CT, the visual criteria performed identically excellent as compared with an optimal SUV_max_ ratio cut-off of 1.44 after ROC curve evaluation. Clinical disease severity did not correlate with the size of the SUV_max_ ratio.

Only a few case reports have been published regarding the use of 68Ga-DOTANOC in the prediction of focal CHI, and with variable success [24, 45]. The wider availability of 68Ga-DOTANOC could argue for the use of this tracer, but the need of a systematic evaluation of the performance of 68Ga-DOTANOC has not been met until now.

Our optimal SUV_max_ ratio cut-off in 18F–DOPA PET to predict focal CHI was 1.44. To the best of our knowledge, this is the first attempt to classify an SUV_max_ ratio cut-off in CHI by the use of the ROC. Others have advocated a cut-off ratio between 1.2 [22] and 1.5 [17, 19, 20], either with SUV nominator and denominator values measured as mean SUV (SUV_mean_) [17, 22], or as SUV_max_ [19, 20].

Ribeiro *et al.* [21] calculated the SUV ratio using the SUV_mean_ of the ROI divided by the SUV_mean_ of the pancreas. They found an average (range) SUV ratio of 1.44 (1.2–1.8) in 14 patients with focal CHI. The average value of 1.44 was exactly the same as our optimal cut-off, but far below our median of 1.72, and SUV_mean_ ratios cannot be directly compared to SUV_max_ ratios.

To the best of our knowledge, the current study is the first blinded comparison of the visual judgment *vs*. an SUV ratio cut-off in CHI patients. The test performance of the 18F–DOPA PET/CT was identical when analyzed visually and when the optimal SUV_max_ ratio cut-off was applied. Others have advocated for a visual judgment [15, 18, 21], but only one study reported a blinded evaluation [15]. In practice, a combination of all available data may be helpful in the interpretation of PET/CT scan results, but this will bias the assessment and the diagnostic tool performance. The blinded SUV_max_ ratio cut-off 1.44 should be validated in another population with the same methodology [46]. Our test performance results of 18F–DOPA PET were in line with the results of a meta-analysis [47], which found a pooled AUC of 0.95, a sensitivity of 0.89 (0.81–0.95) and a specificity of 0.98 (0.89–1). The meta-analysis did not differ between visual or quantitative analysis of the PET scans or the method to obtain SUV ratios. Kühnen *et al*. have in a more recent, unblinded study found a sensitivity for predicting focal CHI of 100% [48]. However, in 3 of their 32 patients, the extent of giant focal lesions was severely underestimated by 18F–DOPA PET. In our series, no giant focal lesions were present, which is why the performance of 18F–DOPA PET *vs*. 68Ga-DOTANOC PET could not be estimated for such lesions.

The combination of 18F–DOPA PET and CT (or MRI) is an advantage for obtaining superimposed pictures of both modalities as guidance for the surgeons. We, as others [17, 19], stress the use of diagnostic CT when a focal lesion is suspected. In fact, one of our two failures in predicting the location of the focal CHI lesion occurred in one of our first patients, in whom diagnostic CT as an exception was not performed, which is why a tail focus was impossible to discern from kidney uptake. The other blinded localization failure by 18F–DOPA PET/CT correctly located the lesion in the pancreatic head, but failed to identify that the lesion was in fact ectopic and situated in the duodenal wall, extending into the pancreas. In fact, the possibility of an ectopic lesion was raised in the nuclear medicine report, as seen after unblinding. Focal ectopic lesions are rare and this has led to extensive redundant pancreatic resections before the introduction of 18F–DOPA PET/CT [49, 50]. For 68Ga-DOTANOC PET/CT, caution should be raised to not interpret labeling in an accessory spleen as an ectopic or pancreatic tail focal lesion, as 68Ga-DOTANOC PET shows physiological labeling in splenic tissue in contrast to 18F–DOPA PET. No such errors were seen in our blinded study, nor in the clinical setting.

We took the opportunity to evaluate the glucose demand and the hyperinsulinism diagnosis during a short hypoglycaemia after discontinuation of medication. This procedure allowed us to reveal that the glucose infusion rate off-medication, as well as other biochemical parameters, did not correlate with the size of the SUV_max_ ratio in focal CHI. This is in keeping with a case report of a child with a high SUV_max_ (no ratio given) and a visually clear focal lesion both before and after clinical remission [51].

Not all CHI centers use time series [20–22] or general anesthesia [19, 21, 22] for 18F–DOPA PET/CT as we did. Our time series protocol revealed that the time of maximal SUV varied from 10 to 60 min in 18F–DOPA and 30 to 60 min in 68Ga-DOTANOC scans, suggesting the advantage of a time series at least in 18F–DOPA PET, where intra-pancreatic bile duct tracing may occur. General anesthesia was preferred in most of our patients with prolonged need for an unmoving child.

## Strengths and limitations

Strengths of our study included the blinded analysis by two researchers with different experiences and a third blinded researcher to decide any disagreements. The high kappa and ICC values showed that the PET/CT scan estimates were robust and that knowledge of genetic and clinical details was not mandatory for an excellent diagnostic performance. Moreover, we used SUV_max_, which is more reproducible than SUV_mean_ [52], and bias from a single or very few pixels was minimized by use of an ROI shaped as a sphere. Lastly, we used both histology and genetics as gold standards, but calculations for the histology gold standard alone did not change the estimates.

Limitations included the retrospective nature of the study, potential misclassification of focal *vs.* diffuse CHI based on genetic results in patients not subjected to surgery, and the earliest used dHPLC method in the genetic analysis, believed to be of lower quality [53].

## Conclusion

In conclusion, blinded 18F–DOPA PET/CT interpretation was excellent in predicting focal CHI and localizing the lesion. 68Ga-DOTANOC PET had a lower point estimate of the ROC AUC and superiority of 18F–DOPA PET/CT was seen, which is why further use of 68GA-DOTANOC PET/CT in predicting focal CHI is discouraged.

## Ethics

The study was approved by the Danish Health Research Ethics Committee (protocol no. 48572), and the Danish Data Protection Authority (protocol no. 2015–41-3867). For this type of study, formal consent was not required.

## Electronic supplementary material

ESM 1 (DOCX 167 kb)
