# Supplementary material for: 18F-DOPA PET/CT and 68Ga-DOTANOC PET/CT scans as diagnostic tools in focal congenital hyperinsulinism: a blinded evaluation
Source: Eur J Nucl Med Mol Imaging. 2017 Nov 8;45(2):250–61. doi: 10.1007/s00259-017-3867-1 (PMC5745571; doi:10.1007/s00259-017-3867-1)
Supplement: Supplementary file 1 — (DOCX 167 kb) [file 259_2017_3867_MOESM1_ESM.docx]

Online Resource:

**CD Christiansen et al. 18F-DOPA PET/CT and 68Ga-DOTANOC PET/CT scan as diagnostic tools in focal congenital hyperinsulinism: A blinded evaluation**

## Table 1 ICC values for 68Ga-DOTANOC PET/CT

| Measurement | ICC | 95% CI |
| --- | --- | --- |
| SUV-max, pancreas  SUV-ratio*  SUV, liver **  maximal SUV-ratio (pancreas/liver)**  SUV, spleen **  maximal SUV-ratio (pancreas/spleen)** | 0.953  0.830  0.965  0.954  0.995  0.988 | 0.882 - 0.982  0.609 - 0.932  0.928 - 0.983  0.885 – 0.983  0.989 – 0.997  0.968 – 0.995 |

*Measurement of the SUV-ratio in the pancreas was performed as for 18F-DOPA PET/CT.

**Measurement of the SUV-max was obtained in a homogenous area of the spleen and liver. In the liver, SUV-max was obtained in an area without visible bile ducts. The liver and spleen ratios were calculated by SUV-max obtained in a suspected focal areas of the pancreas divided by the SUV-max of the liver or spleen. The values were obtained at times 10, 30, 60 min after injection. The highest SUV-ratio was used and location was noted.

# **Table 2** Test performance of 68Ga-DOTANOC PET using different calculations

Table 1. a) Patients with histology or genetics as gold standard

| Method | No* | Sensitivity  (95% CI) | Specificity  (95% CI) | PPV  (95% CI) | NPV  (95% CI) | Location** (%) |
| --- | --- | --- | --- | --- | --- | --- |
| visual | 16 | 0.78  (0.45-0.94) | 0.86  (0.49-0.97) | 0.88  (0.53-0.98) | 0.75  (0.41-0.93) | 7/7  (100) |
| Pancreas SUV_max_ cut-off = 6.77 | 16 | 0.67  (0.35-0.88) | 0.71  (0.36-0.92) | 0.75  (0.41-0.93) | 0.63  (0.31-0.86) | 6/6  (100) |
| Intrapancreatic SUV ratio: cut-off = 1.60 | 16 | 0.67  (0.35-0.88) | 0.86  (0.49-0.97) | 0.86  (0.49-0.97) | 0.67  (0.35-0.88) | 6/6  (100) |
| Pancreas/liver SUV ratio:  cut-off = 2.90 | 16 | 0.67  (0.35-0.88) | 0.71  (0.36-0.92) | 0.75  (0.41-0.93) | 0.63  (0.31-0.86) | 5/6  (83) |
| Pancreas/spleen SUV  ratio: cut-off = 0.66 | 16 | 0.89  (0.57-0.98) | 0.57  (0.25 – 0.84) | 0.73  (0.43 - 0.90) | 0.80  (0.38-0.96) | 6/8  (75) |

Table 1. b) Patients with histology as gold standard

| Method | No* | Sensitivity  (95% CI) | Specificity  (95% CI) | PPV  (95% CI) | NPV  (95% CI) | Location** (%) |
| --- | --- | --- | --- | --- | --- | --- |
| visual | 14 | 0.78  (0.45-0.94) | 0.80  (0.38-0.96) | 0.88  (0.53-0.98) | 0.67  (0.30-0.90) | 7/7  (100) |
| Pancreas SUV_max_ cut-off = 7.73 | 14 | 0.56  (0.27-0.81) | 0.60  (0.23-0.88) | 0.71  (0.36-0.92) | 0.43  (0.16-0.75) | 5/5  (100) |
| Intrapancreatic SUV ratio: cut-off= 1.60 | 14 | 0.67  (0.35-0.88) | 0.8  (0.38-0.96) | 0.86  (0.49 – 0.97) | 0.57  (0.25-0.84) | 6/6 (100) |
| Pancreas/liver SUV ratio:  cut-off = 2.90 | 14 | 0.67  (0.35-0.88) | 0.8  (0.38-0.96) | 0.86  (0.49 – 0.97) | 0.57  (0.25-0.84) | 5/6  (83) |
| Pancreas/spleen SUV  ratio: cut-off = 0.83 | 14 | 0.67  (0.35-0.88) | 0.8  (0.38-0.96) | 0.86  (0.49 – 0.97) | 0.57  (0.25-0.84) | 5/6  (83) |

*number of patients with a scan and a gold standard

**correct located/correct focal identification

# **Figure 1** ROC – 68Ga-DOTANOC PET


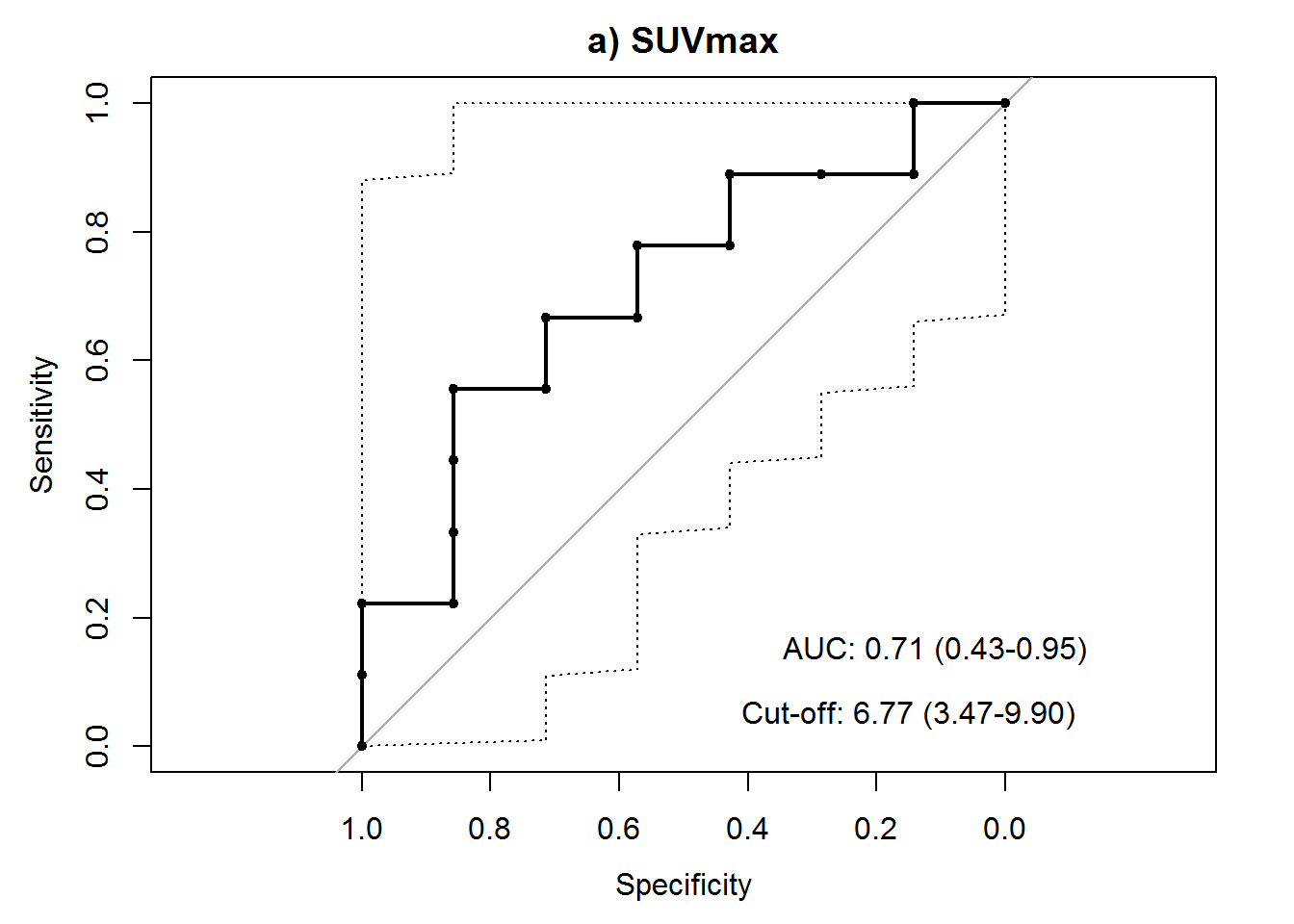

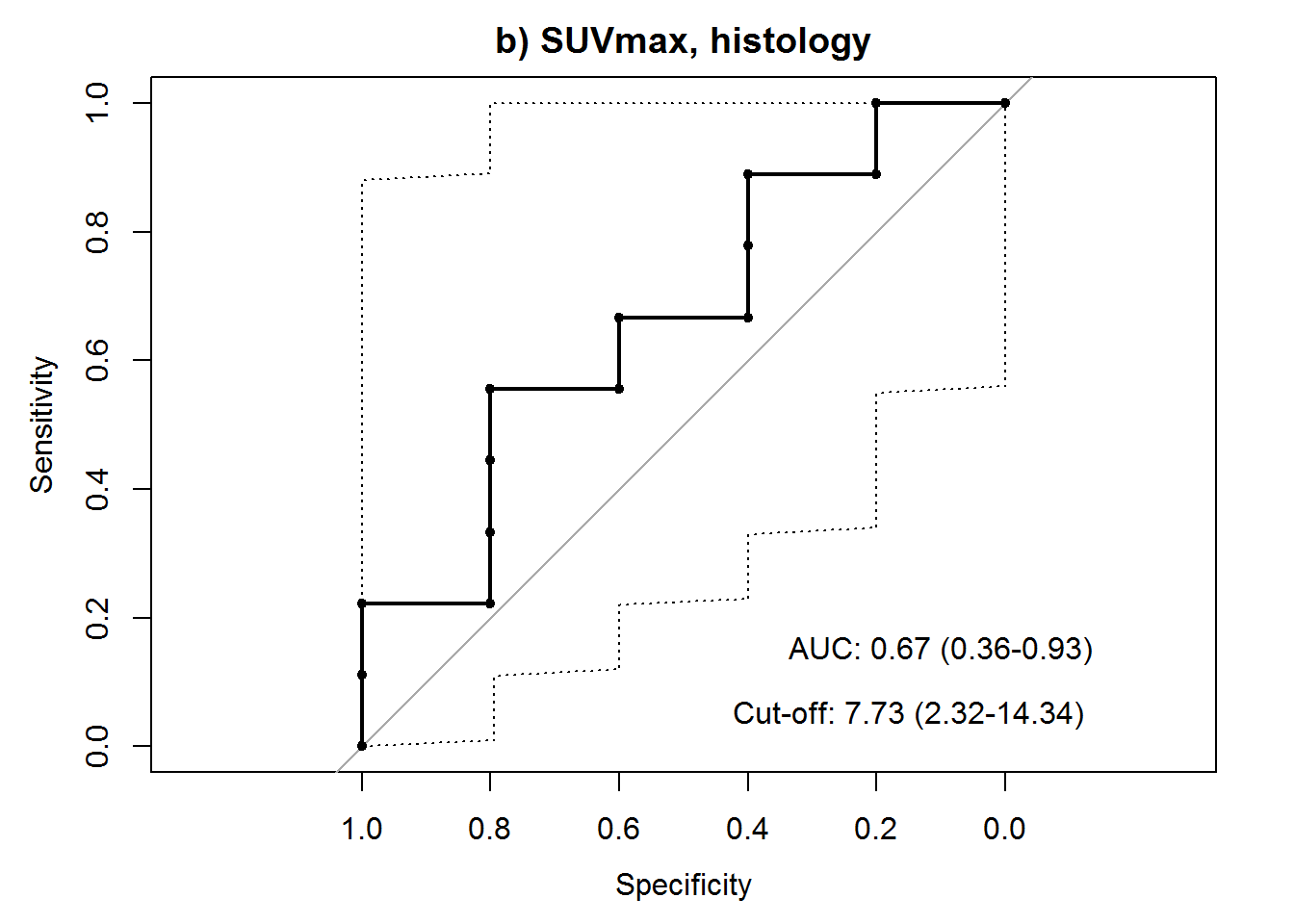

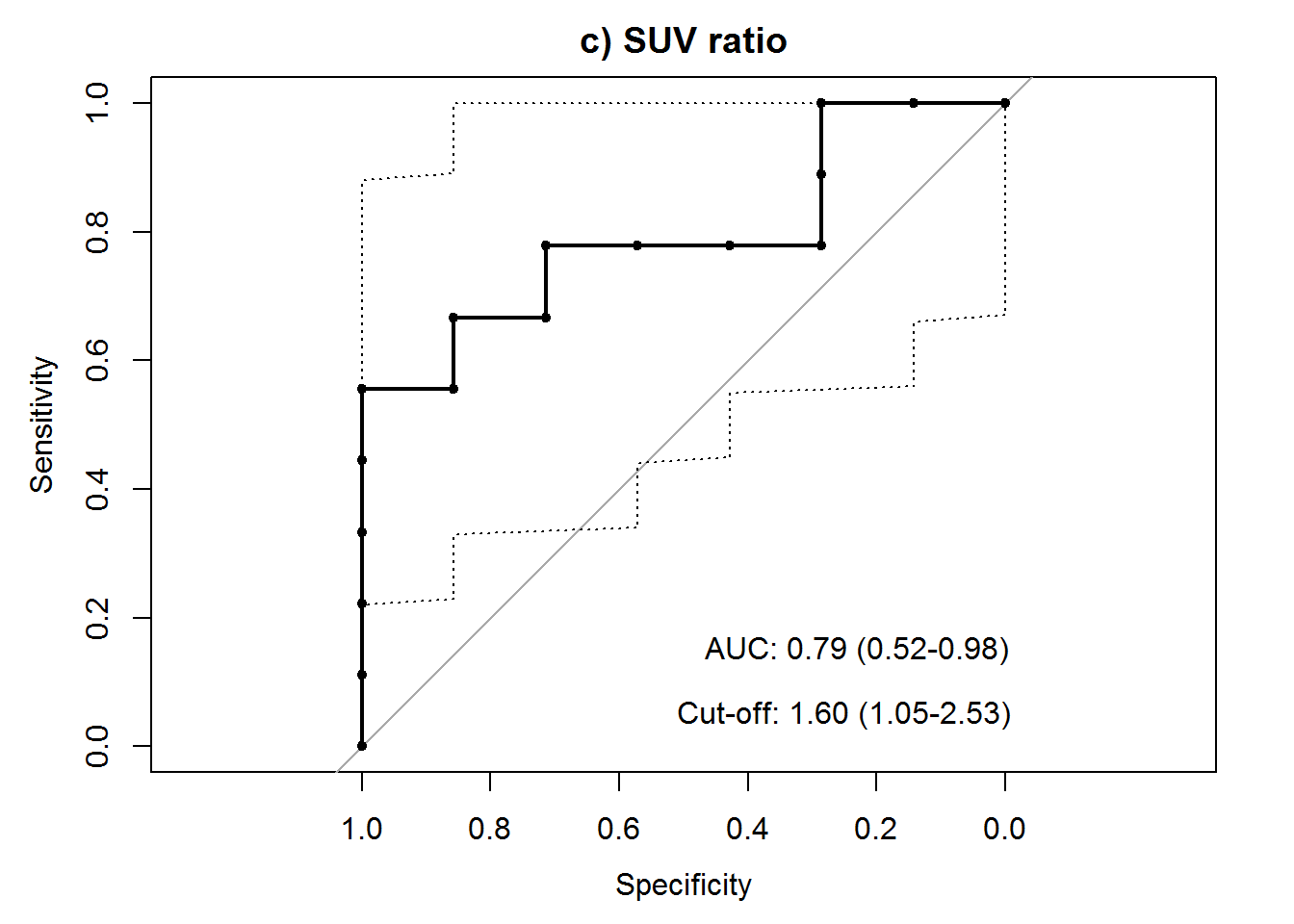

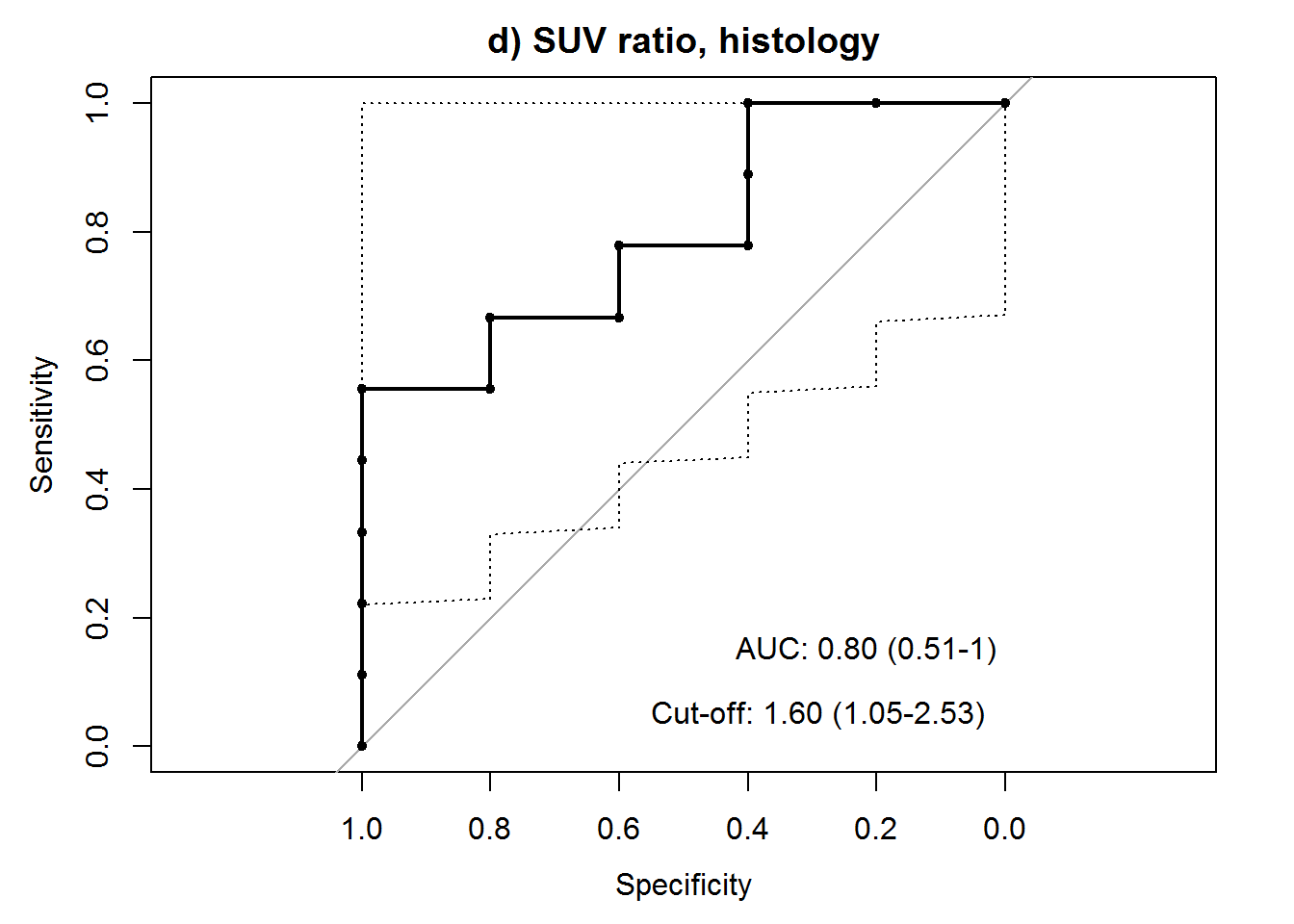

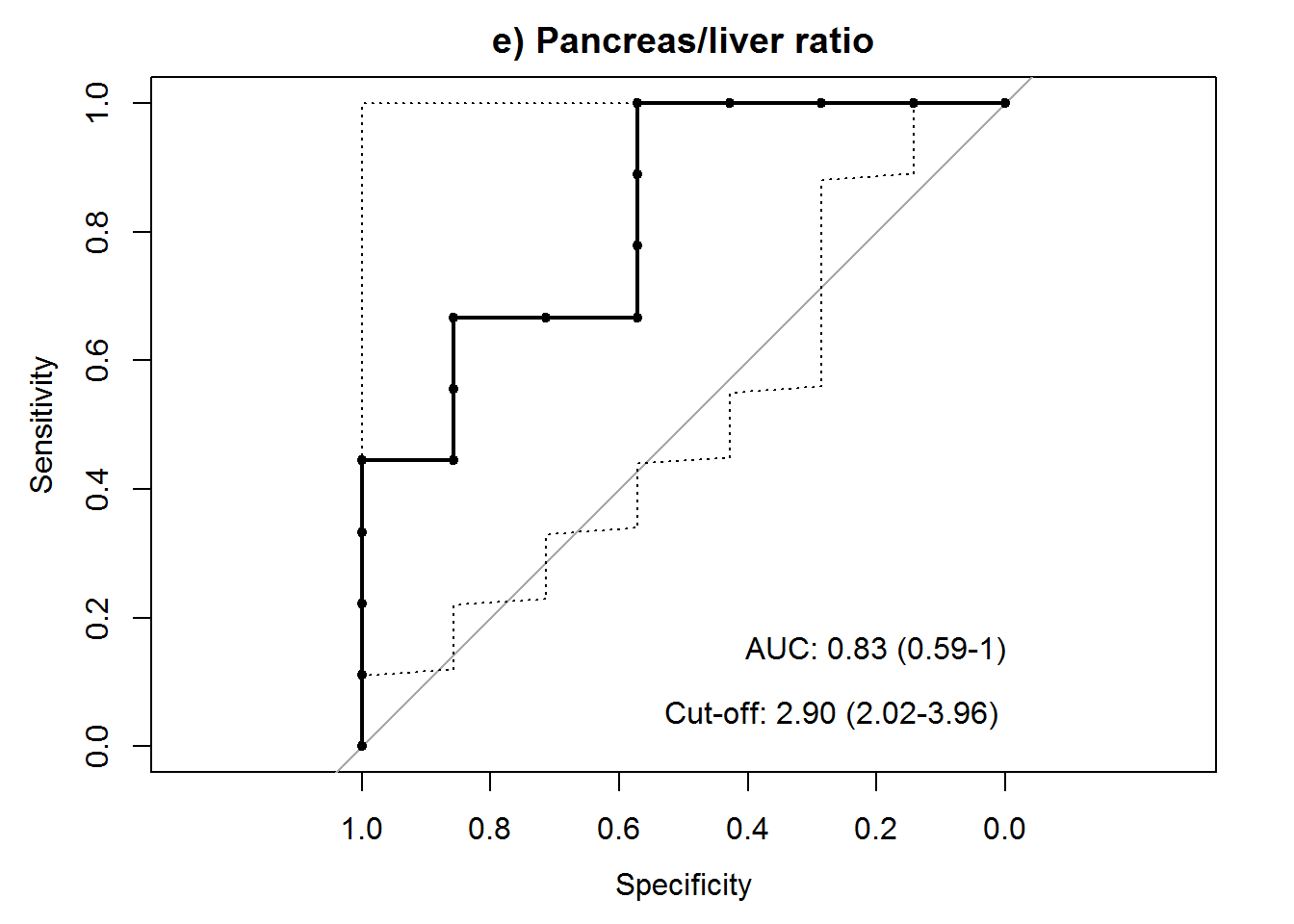

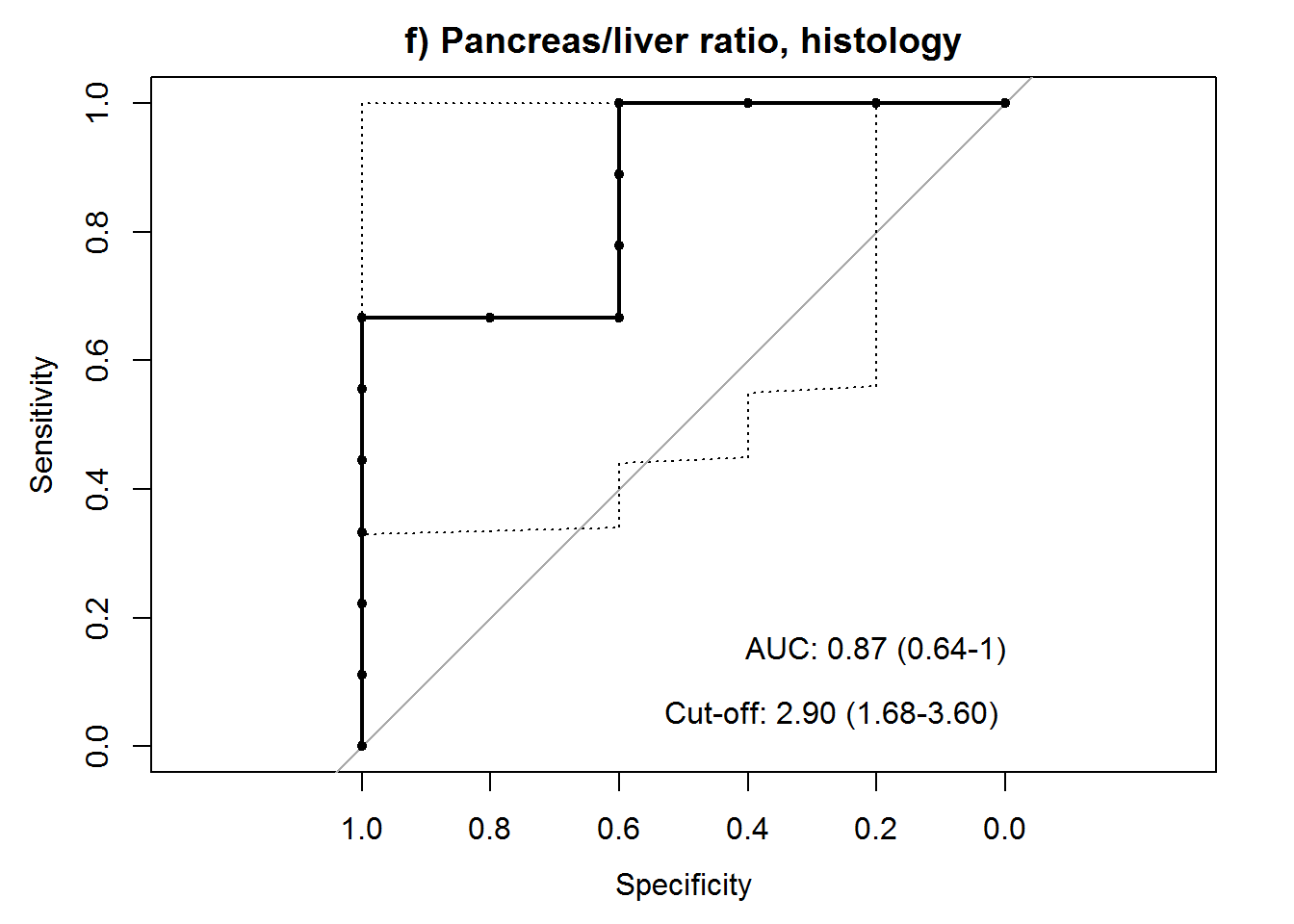

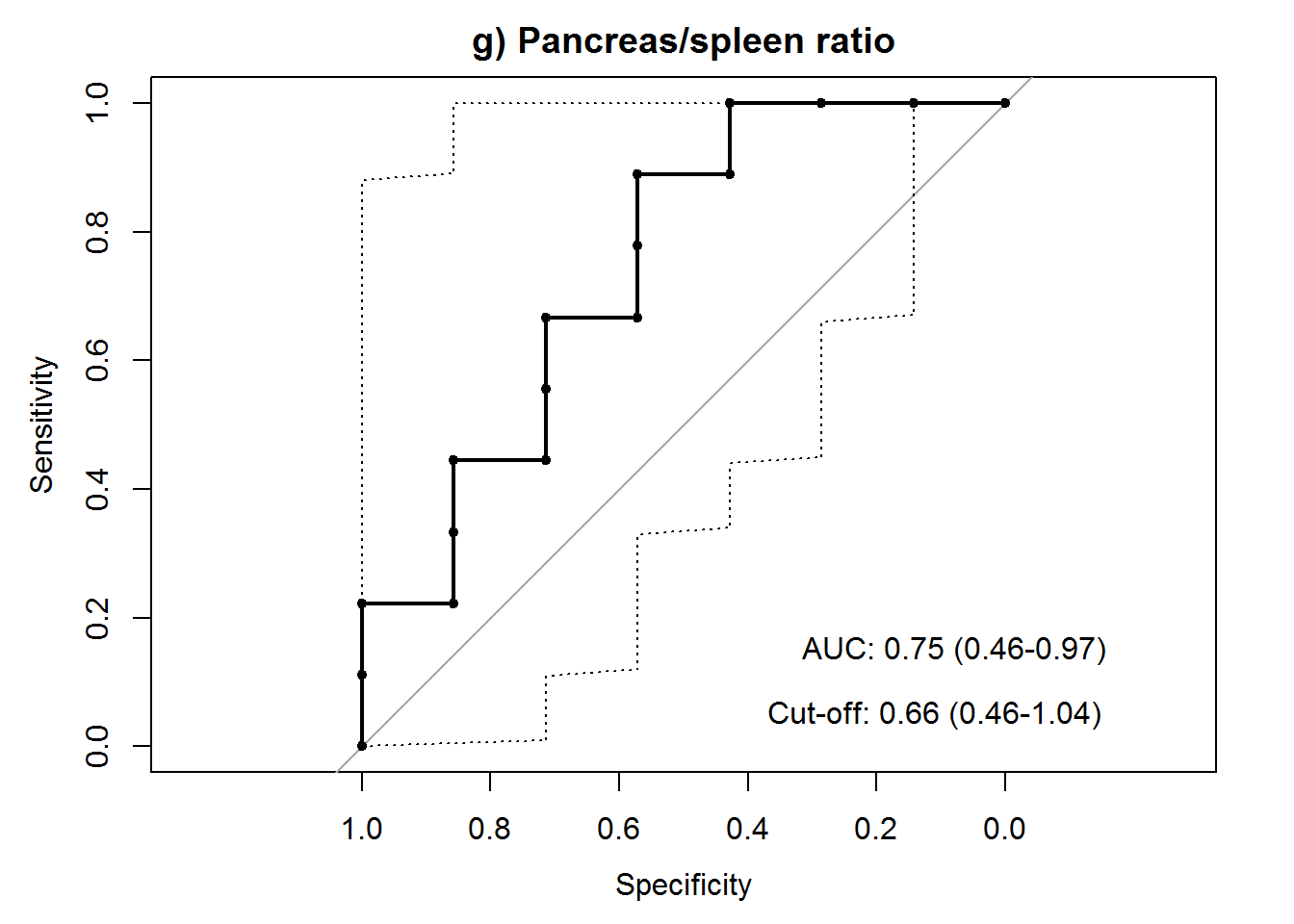

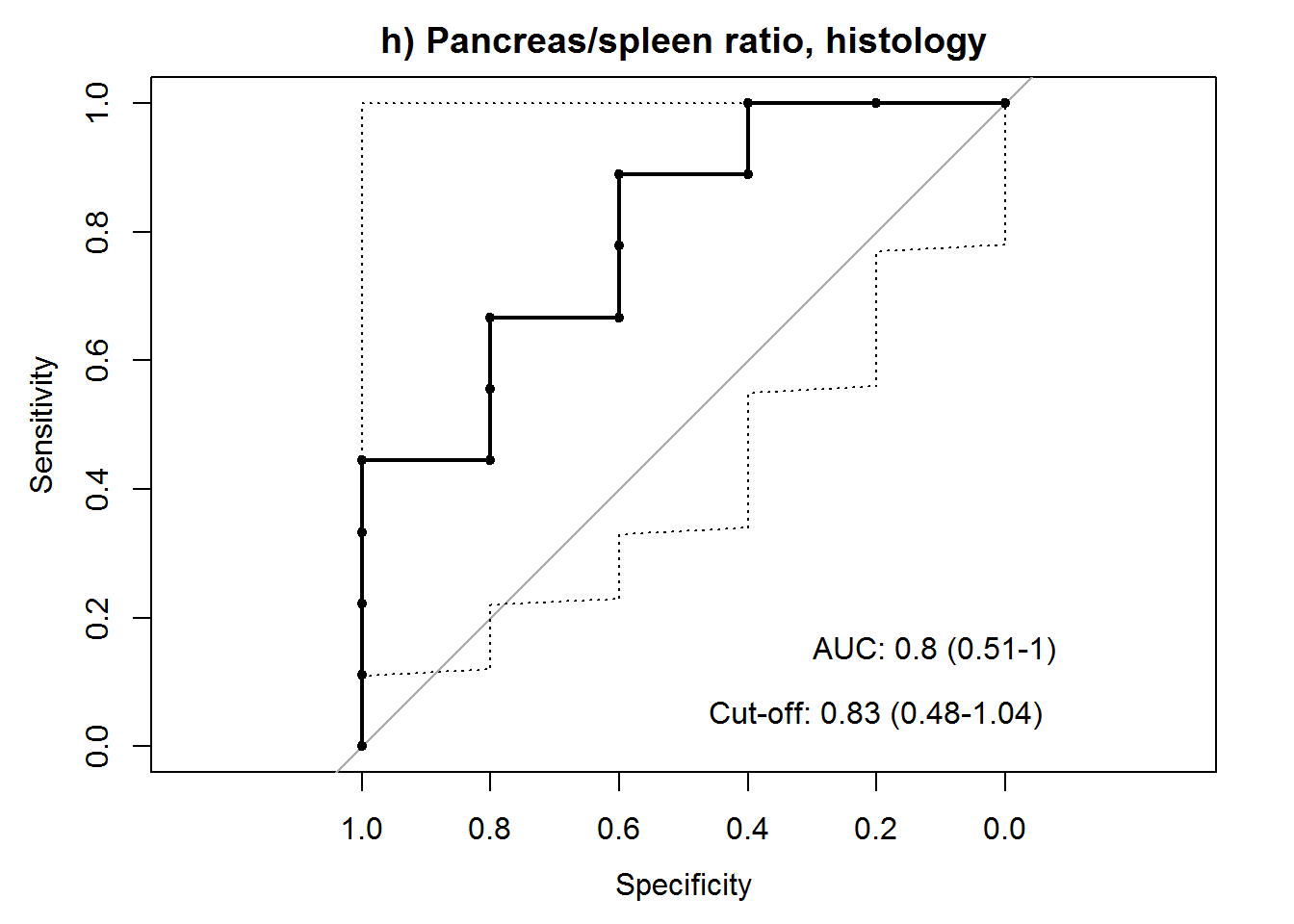


The dotted line indicates the 95% CI of the specificity, calculated by a bootstrap of 10000.

Histology indicates that it was used as singular gold standard
